# Stress and Emotion Open Access Data: A Review on Datasets, Modalities, Methods, Challenges, and Future Research Perspectives

**DOI:** 10.1007/s41666-025-00200-0

**Published:** 2025-06-18

**Authors:** Aleksandr Ometov, Anzhelika Mezina, Hsiao-Chun Lin, Otso Arponen, Radim Burget, Jari Nurmi

**Affiliations:** 1 Wireless Research Center, Tampere University, Tampere, Finland; 2 Department of Telecommunications, Brno University of Technology, Brno, Czechia; 3 Tampere University Hospital, Tampere, Finland

**Keywords:** Emotion, Stress, Recognition, Detection, Dataset, eHealth, Wearable, Open access, Review

## Abstract

Remote continuous patient monitoring is an essential feature of eHealth systems, offering opportunities for personalized care. Among its emerging applications, emotion and stress recognition hold significant promise, but face major challenges due to the subjective nature of emotions and the complexity of collecting and interpreting related data. This paper presents a review of open access multimodal datasets used in emotion and stress detection. It focuses on dataset characteristics, acquisition methods, and classification challenges, with attention to physiological signals captured by wearable devices, as well as advanced processing methods of these data. The findings show notable advances in data collection and algorithm development, but limitations remain, e.g., variability in real-world conditions, individual differences in emotional responses, and difficulties in objectively validating emotional states. The inclusion of self-reported and contextual data can enhance model performance, yet lacks consistency and reliability. Further barriers include privacy concerns, annotation of long-term data, and ensuring robustness in uncontrolled environments. By analyzing the current landscape and highlighting key gaps, this study contributes a foundation for future work in emotion recognition. Progress in the field will require privacy-preserving data strategies and interdisciplinary collaboration to develop reliable, scalable systems. These advances can enable broader adoption of emotion-aware technologies in eHealth and beyond.

## Introduction

The rise of wearable technology and mobile sensors is greatly transforming emotion recognition and eHealth monitoring [1]. These technologies have opened an opportunity to unobtrusively collect large datasets that offer deep insights into physiological and behavioral patterns related to a wide range of emotions [2]. As an inner research direction, emotion detection is becoming a crucial part of future eHealth systems, with the potential to improve patient care significantly [3, 4]. Healthcare providers can offer more personalized and effective treatments by accurately identifying and responding to patients’ emotional states. For example, systems that detect emotions can help manage chronic conditions by monitoring patients’ emotional well-being [5], provide more insight on sleep quality [6], deliver timely interventions [7], and detect subclinical disorders early [8].

Integrating emotion detection into eHealth/telehealth platforms, which the COVID-19 pandemic has especially boosted, strongly enhances patient engagement and satisfaction by addressing emotional needs during virtual consultations [9] as well as brought the attention to the need of industrial monitoring or workers, especially operating in harsh conditions [10, 11]. This, in turn, is important for managing mental health, where understanding emotional states is key to accurately diagnosing symptoms and further providing necessary and appropriate treatments [12].

Notably, emotion detection is important not only for individual care but also for public health initiatives. Systems that recognize emotions can help detect mental health issues early, enabling preventive measures and reducing the burden on healthcare systems [13]. They can also support remote monitoring, making healthcare more accessible to underserved populations [14]. As the global population ages and chronic diseases become more common, the need for emotion-sensitive eHealth solutions will keep growing [15].

However, collecting and, especially, publishing emotion-related data is an exceptionally complex process because emotions are subjective and influenced by many aspects, including individual differences, cultural contexts, and operational scenarios [16]. This complexity makes it hard to create standardized methods for collecting and annotating emotion data [17]. In particular, stress is a multifaceted affective state, which is not a discrete emotion but rather a state emerging from intersections between valence, arousal, and dominance [18, 19]. Few other emotions (e.g., fear or frustration) and mental disorders (e.g., anxiety or depression) share co-occurring states [20]. Therefore, from affective computing perspectives, recognizing stress-related emotional states requires a multidimensional approach, which is impacted by numerous elements (e.g., personal subjective experiences, contextual and cultural differences), to provide a more nuanced interpretation of stress [21]. Additionally, the ever-changing nature of emotions means continuous monitoring and real-time analysis are required, adding to the difficulty and pushing the need for personalized machine learning (ML) and deep learning (DL) solutions to be developed [22, 23]. Despite these challenges, advances in sensor technology and data analysis have improved the accuracy of capturing and interpreting emotional signals [7].

According to [24], different approaches to collecting emotion data. These data could be combined under a generic umbrella of modality term. Multiple modalities are often involved when the person expresses emotions, whether explicitly, e.g., as through facial expression and speech, or implicitly, e.g., via text or images, ranging from self-reports to physiological and behavioral modalities. On the one hand, while self-reported data is useful, it can be biased due to its subjective nature based on human annotation. In this case, expert opinion and feedback are required. Simultaneously, devices designed to collect physiological signals, e.g., heart rate (HR) monitors and skin conductance sensors embedded in smart wearables, provide objective data but need careful calibration and interpretation [25]. Behavioral modalities, including facial recognition and voice analysis, offer additional insights; however, it also raises privacy, General Data Protection Regulation (GDPR), and informed consent [9]. Merging multiple data sources, e.g., creating a multimodal approach, is increasingly seen as a more holistic and effective way to capture the full complexity of emotional experiences [26].

Moreover, even traditional aspects of processing and storage of data remain major concerns because most of the biometric data could be considered sensitive and personal [9]. Ensuring informed consent and protecting data from unauthorized access is crucial to maintaining trust and complying with regulations [13]. There are also ethical considerations about the potential misuse of emotional data, e.g., for surveillance or manipulation. Establishing clear regulatory frameworks and ethical guidelines is essential to address these issues and ensure the responsible use of emotion detection technologies [14].

Besides, the European Parliament’s study on biometric recognition and behavioral detection advocates for explicit regulation of Emotion Recognition System (ERS)s under the European Union (EU) Artificial Intelligence Act (AIA) [27]. It recommends integrating transparency obligations [28], ensuring individuals are informed about ERS usage, and restricting decisions based solely on biometric data [29]. The study highlights the need for automated consent management and emphasizes that ERS should only be used when strictly necessary, with explicit consent, or for legally justified purposes. These recommendations align with GDPR [30] but may lead to significant reformulation on how unobstructed emotion recognition could be integrated into real eHealth systems. Therefore, it can be concluded that the integration of emotion recognition into the factual healthcare domain is still far from being implemented. Yet, regulative documents are laying the possibility for this to occur.

Notably, the emotion recognition has been extensively studied in recent years followed by numerous research papers addressing various methodologies and frameworks. However, the restricted accessibility of the datasets used in these studies continues to be a significant obstacle in this field. Most of the existing works fail to share their collected datasets, often due to restrictions related to privacy, proprietary concerns, or institutional limitations. Consequently, these datasets remain closed to the original research groups, preventing broader validation, comparison, and replication of results across different studies. This lack of data openness significantly limits the reproducibility and generalization of emotion recognition models, posing a barrier to further advancements in the field.

Despite these challenges, a few datasets for emotion detection research are available in open access. These datasets differ in size, scope, and the types of modalities they capture, providing valuable resources for training and testing emotion recognition models, helping to advance the field. Addressing these gaps can lead to the development of more accurate and robust emotion detection systems that better support eHealth applications [13].

This paper aims to explore the datasets used in recent research to systematize their characteristics, collection methods, and potential limitations. Specifically, we address three main research questions:

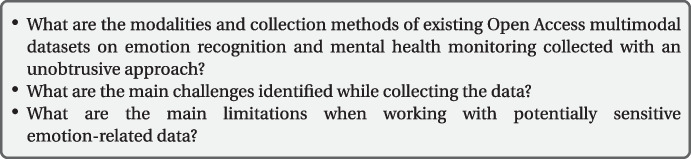



To answer these questions, we analyzed (claimed to be) open access datasets from recent research works, focusing on the types of modalities used, the data collection scenarios, and the challenges faced. We also assessed the availability and accessibility of the datasets for the research community. By examining these aspects, we aim to provide a thorough overview of the current state of emotion detection datasets and identify gaps and opportunities for future research. The factual open accessibility was the main selection criterion of those works, limiting the research community to only nine fully open access sources that became the main subject of this review.

The rest of the paper is structured as follows. First, the manuscript outlines the methodology used for the dataset search in Section 2. Next, Section 3 provides an overview of approaches to search existing datasets, including the main modalities used, and strategies to request the data behind a registration wall. Further, Section 4 details the factually open access datasets, data collection approaches, various populations (demographic and other coverages), and technical aspects of the data itself from storage and processing perspectives. Section 5 outlines the technical challenges identified and solutions proposed (as per dataset collectors). Section 6 delves into the limitations of data from other perspectives. The last section provides a summary of all findings. Appendixes detail the approach to sources screening, as well as provide more details on the fully open access datasets’ populations and modalities.

## Methodology

**Fig. 1:**
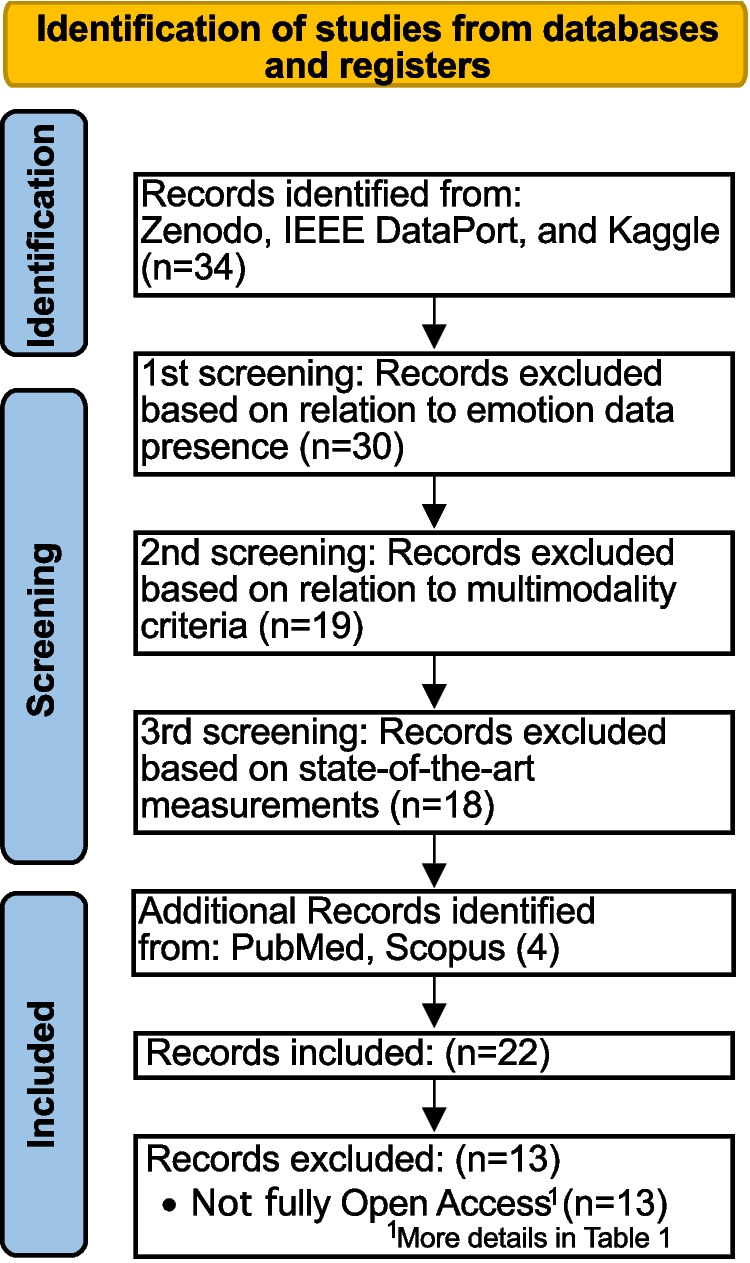
Datasets screening methodology

To identify relevant datasets for this study, we conducted a narrative search across multiple online repositories, including Zenodo, IEEE DataPort, and Kaggle (see Fig. 1). Additionally, we extended the search by including research papers that introduced new datasets and provided publicly available links to their sources. The Scopus-generalized search query used was “{{depression OR stress OR emotion OR anxiety OR mental} AND {detection OR classification} AND data*}” focusing on emotional and mental health assessment datasets through a multimodal approach. As previously mentioned, stress is a multifaceted emotional state, and we include anxiety and depression into the search due to they share over-lapping symptoms [31, 32]. Notably, it was also adjusted for different sources. Thus, data from wearable devices (potentially supplemented with other modalities, e.g., self-reports) formed the majority of the datasets. The methodology adopted the approach of the adapted the Preferred Reporting Items for Systematic reviews and Meta-Analyses (PRISMA) guidelines. More details about the methodology are available in Appendix A.

The initial search resulted in 34 datasets. At first, the main exclusion criterion was defined as to “data accessibility upon request or available partially; factually restricted access; availability only by email request;” “statement to be accessible but not in practice.” After the revision, the main exclusion criterion was full open access to the data. Yet, we decided to still list those initially excluded ones in Table 1, highlighting those in the column “Access.” It also allows the reader to have a broader understanding of the limitations.

**Table 1:** Emotion-related open access datasets as of 08.09.2024: $\looparrowright$, data accessible upon request or available partially;  , restricted access;  , available by email request;  , stated to be accessible but not in practice

Refs.	Link	Name: wearables/sensors	Types of emotions	Modalities	Major challenge	Access
[33, 55]	url [46]	**StudentLife**: Smartphone	Depression, stress, loneliness	ACC (running, walking, sedentary), location (GNSS), phone call frequency and duration, self-assessment	Data privacy concerns, limited participant profiles, and small dataset; no biometric data	
[56]	url	**MODMA**: 128-Ag/AgCl electrodes elastic Cap (HydroCel Geodesic Sensor Net, HCGSN), and a microphone	(In)congruent conditions: happy, sad, and fear	EEG and recordings of spoken language data	Limited number of samples, limited number of mental illnesses	
[57]	url	**SEED**: An electrode cap and a camera	Positive, neutral, and negative emotions	EEG, eye-tracking	Limited number of emotion classes; small number of participants; limited modalities	
[58]	url	**DREAMER**: The Emotiv EPOC headset and the SHIMMER ECG sensor	Valence, arousal, and dominance	EEG, ECG, self-assessment	Controlled enlivenment may not reflect the real-world conditions	
[59, 60]	n/a	**imec**: chillband, mobile app, GoPro camera	Non-, light, and high stressed	ACC, Galvanic Skin Response (GSR), SKT, physical activities (sitting, standing, walking, sedentary, cycling), sleeping time and duration	Incorporating context data;	
[19]	url	**Tesserae**: Garmin Vivosmart 3, a Zephyr chest strap, Bluetooth Beacons and key-fob, smartphone	Binary stress classes	ACC, HR, HRV, GNSS, phone usage, sleep duration and quality, environmental conditions, self-assessment	Long-term data collection, unable to model the temporal dynamics during a single day due to the low temporal resolution of stress labels	
[61]	url	**TILES**: Fitbit, smartphone, smart shirt, the Owl-in-One data hub	Binary stress classes	ECG, breathing patterns, sleep quality, activity patterns, location, audio recording, self-assessment	A combination of location, speech features, and circadian rhythmcontextual information is needed in the *hospital* setting	
[34, 62]	url [47]	**WESAD**: Polar M600 smartwatches, smartphone (Android)	Binary stress classes	Lab: ECG, EDA, RSP, SKT, ACC. Field: IBI, HR, ACC, phone usage, GNSS, self-assessment	Ensuring accurate and continuous data collection in varying environments. Adapting to individual differences in stress responses	
[35]	url [63]	**LaborCheck**: Fitbit	Three stress levels: low, medium, high	HR, ACC	Variability in physical activities	
[36, 64]	url [50]	**StressDetection**: Smartphone	Binary stress classes	HR, phone usage (touchscreen), gravity, ACC, GYRO, self-assessment	Suffer from sufficient data extraction; no biometry	
[65]	n/a	**Boğaziçi**: Samsung Gear S1 and S2 smartwatches, Empatica E4	Low, medium, high	IBI, HRV, EDA, ACC, SKT	Limited to the NASA-TLX PerceivedWorkload and Perceived Stress, need to incorporate other stress measurement scales.	
[66]	url	**K-EmoCon**: NeuroSky MindWave Headset, Empatica E4, Polar H7, a head-mounted camera	20 categories of emotions	EEG, ECG, EDA, BVP, IBI, HR, ACC, SKT	Limited sample size, short duration, non-accurate measurements by off-the-shelf devices	
[67]	n/a	**UC**: Smartphone (iOS/Android), Samsung Galaxy wristwatch	Depression ratings	HR, HRV, sleep duration, ACC, step count, self-assessment (mood and lifestyle rating)	Difficult to access large and standardized database of already treated patients in a clinical setting	
[37, 68]	url [44]	**MoodAware**: Empatica 4	Binary stress classes	BVP, EDA, ACC, SKT	Variability in daily life conditions; quite limited in terms of factors	
[41]	url [51]	**GLOBEM**: Smartphone, Fitbit wrist worn	Binary depression classification	Location, phone usage (screen status), Bluetooth, call logs, WiFi, physical activities (step counts, sedentary episodes), sleep duration and quality	Privacy concerns; quality of data can vary due to differences in devices usage; no biometry	
[69]	n/a	**Ellipsis**: Smartphone	Binary classification on anxiety and depression	Voice samples	Limited to older subjects, semantic analysis of speech, and binary classification. Difficult to reflect real-world use.	
[38, 70]	url [49]	**ADARP**: Empatica E4	Binary stress classes	EDA, ACC, SKT, BVP, HR, user input	Imbalanced dataset can lead to biased ML models, which may not perform well in real-world scenarios	
[39, 71]	url [53]	**EmpaticaE4Stress**: Empatica E4	Stress levels	BVP, EDA, SKT, HR, IBI, ACC	Substantial noise due to the different movements, and between tasks subjects might not be completely relaxed; complicated to identify labeled data; not real-life conditions	
[72]	n/a	**Donders**: Empatica E4	Binary: positive and negative moods	HR, skin conductance, self-assessment	High variability in stress responses; Reliability of the wearable tomeasure skin conductance in daily life scenarios	
[73]	url	**MDMER**: Wireless dry electrode EEG device DSI-24, Intelligent wristband ES1	Positive, negative, and mixed emotions	EEG, GSR, PPG, frontal face videos, self-assessment	Limited number of emotion classes: positive, negative, and mixed; subjective self-reports on emotions; Difficult to classify particular emotions among different individuals	
[40]	url [54]	**EmoWear**: ST SensorTile.box, Zephyr BioHarness 3, Empatica E4	Valence, arousal, and dominance	ACC, GYRO, ECG, BVP, RSP, EDA, SKT, self-assessment	Need to manually check the synchronization of event markers with sensor data; Some data are missing for several participants	
[74]	url	**BrainAmp**: EEG system, microphone, camera	**EAV**: Neutral, sadness, anger, happiness, and calm	30-channel EEG, audio, video	Differences in how participants express emotions; Controlled environment.	

**Acronyms**: EEG, ECG, BVP, SKT (SKT and TEMP merged for simplicity), ACC, GYRO, PPG, RSP, IBI, HR, GNSS, HRV, EDA and GSR refer to the same but are kept separately

The filtering process led to the removal of inaccessible datasets or not based on multiple modalities, as well as those based solely on functional magnetic resonance imaging (fMRI) due to classification limitations and insufficient state variability. Additionally, an outdated dataset from 2011 was excluded. After the exclusion process, 18 datasets remained, and additional four datasets containing smartphone data were incorporated to enhance the dataset pool (those were added based on an extended literature search or additional works by the same contributors). This final selection ensures a focus on accessible, recent, and relevant datasets for depression and emotion detection using wearable technologies.

Notably, we included all relevant open access datasets without specific requirements on sample size, annotation quality, sensor modalities, or geographic diversity due to the limited number of available datasets on emotion, stress, and mental health.

## Related Research Work Overview

Most of the openly available datasets are accompanied by a corresponding research paper, as shown in Table 1. The following section elaborates on what those papers are about, as those are not always sole dataset descriptors but rather initial studies on the data analysis.

Before the actual discussion on research papers, the modalities are introduced and discussed to familiarize the reader with them.

### Major Emotion Modalities

The modalities used for the data collection are outlined in Table 1. Due to the multifaceted nature of stress as mentioned earlier, the multimodal emotional data could be collected and classified into the major groups as follows. **Direct physiological indicators**, ElectroCardioGraphy (ECG)/HR, Electrodermal Activity (EDA), Blood Volume Pulse (BVP), Respiration (RSP), Skin Temperature (SKT), Inter-Beat Interval (IBI), Temperature (TEMP), Gyroscope (GYRO); and **Indirect behavioral indicators**, Self-assessment/User Input, Phone Usage, Phone Call Frequency and Duration, Step Count, Sedentary Episodes, Sleep, Global Navigation Satellite System (GNSS), Wireless (Bluetooth Low Energy (BLE), Wireless Fidelity (WiFi)). This classification depicts that both physiological modalities coming from biometric signalsm such as HR, EDA, and RSP, and behavioral data, such as phone usage, step count, and sleep patterns, were selected as important indicators for emotion detection. While physiological modalities provide direct evidence of the body’s response to stress, behavioral modalities offer contextual information that can further enhance the accuracy of stress detection systems. Further, we highlight the modalities present in the identified datasets (sorted based on the frequency of presence in the datasets):

- Accelerometer (ACC) [33–40] is often used in detecting physical activity levels and movement patterns. Stress can manifest through physical restlessness or decreased activity, such as increased sedentary behavior. Accelerometer data can be used to track these patterns, providing indirect insights into stress levels through movement changes.
- Self-assessment/user input [33, 34, 36, 38, 40] or user input through questionnaires or apps allow individuals to assess and report their stress levels subjectively. This is a direct method of understanding stress, often used alongside physiological data to provide a more comprehensive view.
- ECG/HR [34–36, 38, 40], i.e., ECG and HR are directly linked to responses to negative emotions, i.e., those often lead to changes in HR and Heart Rate Variability (HRV), with increased HR and reduced HRV being common indicators of stress or anxiety.
- EDA [34, 37–40] measures the electrical conductance of the skin, which changes in response to sweat. For example, stress triggers the autonomic nervous system, leading to increased sweating. EDA is a widely used physiological arousal and stress measure.
- BVP [37–40] is closely related to HR and can be used to detect changes in cardiovascular activity associated with stress. BVP, often detected via Photoplethysmography (PPG), can indicate changes in blood circulation that correlate with stress responses.
- SKT/TEMP [34, 37–40]. Stress can cause changes in body temperature regulation, often leading to an increase in peripheral skin temperature or changes in core body temperature. Monitoring it can provide indirect insights into stress. It is important to note that SKT and TEMP are not directly correlated with ECG or EDA.
- Location data [33, 34, 41] GNSS modality allows for tracking the patterns in mobility. When individuals are under stress, they may alter their regular routes to their destination by avoiding a certain area, which might induce more stress levels. Hereby, changes in activity location or movement speed may offer insights into stress levels, although it might not be a direct physiological indicator.
- RSP [34, 40] are known to change under a negative change in an emotional state. Shallow, rapid, or irregular breathing can indicate stress or anxiety. Monitoring respiration can provide valuable data for detecting stress.
- IBI [34, 39], the time between consecutive heartbeats, is closely tied to HRV variability. Stress often decreases HRV, which can be detected through IBI measurements. Therefore, monitoring IBI can help assess stress levels based on cardiac autonomic regulation.
- GYRO [36, 40] measures changes in orientation and rotation of the wearable device, often linked to physical movements or postures. While not directly measuring emotion data, abnormal movement patterns or posture changes may indicate, e.g., agitation or nervousness.
- Phone usage [34, 41] could be affected by stress, e.g., through constantly checking the phone, making more phone calls, or sending more text messages. Monitoring phone usage patterns can provide indirect insights into the emotional state, particularly if it correlates with social interactions or emotional responses.
- Step count [41] measurements and monitoring its patterns can be one of the usable modalities, e.g., stress may result in either increased physical activities due to restlessness or decreased activity due to fatigue or avoidance.
- The increase of sedentary episodes [41] is often associated with stress or depression. A person under stress may disengage from physical activity, which can be detected by monitoring periods of inactivity or sedentary behavior.
- Sleep patterns [41] and interrupted sleep are common symptoms of experiencing depression, anxiety, or stress, that often lead to poor sleep quality, insomnia, or irregular sleep cycles.
- Phone call frequency and duration [33] may reveal some signs of stress, especially if it involves emotionally charged conversations to seek social support.
- Wireless communications [41], e.g., Bluetooth or WiFi, can be used to track the location or frequency of social interactions in contrast to traditional GNSS methods [42]. Negative emotional states may influence social engagement or patterns of movement, which can be indirectly assessed through wireless data [43].

To summarize, such a diversity of modalities across datasets underscores the increasing recognition of emotion as a complex, context-dependent phenomenon that cannot be fully captured through a single source of data. The frequent inclusion of both physiological and behavioral indicators highlights a growing trend toward a multimodal approach in emotion detection, aiming to balance objectivity with contextual richness. Such multimodal frameworks not only improve the robustness and generalizability of, e.g., stress inference models but also allow for personalization by capturing the nuanced ways individuals experience and express stress.

### Related Research Papers

Majority of the provided datasets were accompanied by leading research papers, and this subsection outlines those papers and relations with datasets from Table 1 and sorted accordingly.

Can and André [37] investigated the performance of recurrent neural network (RNN) variants, particularly long short-term memory (LSTM) networks, for recognizing daily stress levels using physiological modalities, such as HR and SKT from wearable devices. The study focused on naturalistic conditions, unlike previous lab-based research. Using the publicly available MoodAware dataset [44], the authors evaluated various RNN and convolutional neural network (CNN) combinations with raw data, handcrafted features, and CNN-extracted features. This work’s results showed LSTM achieved up to 95% accuracy with raw features, outperforming models using handcrafted or CNN-based features, highlighting RNN effectiveness for time-series data.

Sanchez et al. [35] developed a predictive model to identify early job stress patterns using Fitbit [45]. The main collected modalities were steps, heart rate, sleep quality, and self-reported stress levels collected from 57 participants. After processing and balancing the data, a dataset of 870 records is analyzed with traditional ML models, e.g., random forest (RF), *k*-nearest neighbors (kNN), Naive Bayes, and AdaBoost. RF delivered the best results, achieving 83% accuracy on training data and 79% on test data, supporting stress management through behavioral and physical activity monitoring.

Thakur [33] studied smartphone usage patterns and sensor data of the StudentLife dataset [46] to predict mental health issues of stress, depression, loneliness, and sleep quality. Specifically, mobility, sociability, and physical activity were proven to be significantly correlated with mental health survey scores. Independent *t*-tests revealed differences in location variance, call duration, and movement patterns between affected and unaffected individuals. Binary classification models achieved an AUC of 82.6% for stress prediction and 74% for depression, showcasing the potential for early mental health issue detection.

Tervonen et al. [34] proposed a stress detection model for older adults using wrist-worn sensors and salivary cortisol in WESAD Dataset [47]. Data from 40 participants undergoing the Trier Social Stress Test [48] was used to measure signals like EDA, BVP, IBI, and SKT. RF classifiers trained with 27 selected features achieved 94% accuracy and an F1 of 0.92. Sensor fusion further improved performance, emphasizing the value of combining multiple physiological signals.

Sah et al. [38] applied CNNs to detect stress in individuals with alcohol use disorder (AUD) using real-world monitoring of the ADARP dataset [49]. Stress detection was improved using a polynomial-time algorithm, identifying EDA as the most indicative modality. Performance peaked with 60-s segments around stress events with 99% accuracy and an F1 of 0.99, while oversampling reduced accuracy to 76.25%, highlighting imbalanced data challenges in real-world scenarios.

Sağbaş et al. [36] developed a real-time stress detection system based on the typing behaviors and smartphone sensors, i.e., GYRO, ACC, gravity, and touchscreen inputs. Feature selection techniques, i.e., ReliefF and genetic algorithms (GA), reduced irrelevant features, improving classification accuracy after applying to the StressDetection dataset [50]. Using kNN and GA-selected features, the system reached 89.61% accuracy and an F1 of 0.9052. The dataset was expanded to include HR data and stress questionnaires, enhancing the robustness of the evaluation.

Xu et al. [41] introduced a longitudinal GLOBEM dataset [51] for passive sensing, collected from 497 users over 700 user-years via wearables. This dataset includes diverse sensor streams and well-being metrics, enabling cross-dataset evaluations and advancing human behavior modeling. The authors benchmarked 18 algorithms for depression detection, highlighting opportunities and challenges to achieve robust generalizability across populations and timeframes.

Campanella et al. [39] explored stress responses in workplace and academic environments using Empatica E4 [52] in Empatica4Stress dataset [53]. Physiological modalities, such as PPG and EDA, were recorded from 29 participants during a personalized protocol simulating real-world stressors. A robust data pipeline ensured high-quality signals, supporting artificial intelligence (AI) model training and stress classification.

Rahmani et al. [40] presented the EmoWear dataset [54] for emotion recognition (ER) using Seismocardiography (SCG) data captured via inertial measurement unit (IMU)s. Data from 49 participants included activities, such as watching emotionally stimulating videos, walking, talking, and drinking. Self-assessments of emotional valence, arousal, and dominance provided comprehensive emotional data. Statistical validation confirmed its utility for studying multimodal emotion analysis and IMU-based context awareness.

In summary, these accompanying studies highlight the broad potential of wearable sensors and multimodal datasets to monitor stress and mental health. They emphasize the value of open access data in enabling personalized, real-time insights for mental health management across diverse populations and environments.

## Datasets Insights

The following section elaborates specifically on the datasets’ content. First, we detail the statistics of the main modalities collected for emotion data. Next, the section focuses on the data collection specifics, followed by notes on demographics and other coverages. Finally, the section provides details about the data itself from data science perspectives.

### Analysis of Content of Selected Datasets

StudentLife dataset [33] comprises three types of data: automatic sensor data, behavioral assessments, and self-reported ecological momentary assessment (EMA) data. Automatic sensing data is collected from smartphones for 10 weeks. This data includes several modalities: activity data, conversation data, sleep data, and location data. Automatic sensing occurs without user interaction, and the data is uploaded to the cloud when the phone is charging and connected to Wi-Fi. The second data type is the EMA data. These assessments are scheduled, managed, and synchronized using the MobileEMA component integrated into the StudentLife app, which also features the Photographic Affect Meter (PAM) for capturing the mood. Finally, there is academic data, which evaluates the student’s academic performance. This dataset can be used to evaluate academic performance and behavioral trends and the impact of workload patterns on daily life.

The WESAD dataset [34] consists of multimodal physiological and motion data collected using two wearable devices: RespiBAN and Empatica E4. It includes high-resolution physiological measurements, such as BVP, ECG, EDA, electromyography (EMG), respiration (RESP), and TEMP, as well as motion data from ACC. The dataset is organized so that each subject has associated information, data from the RespiBAN and Empatica E4 devices, synchronized raw data and labels, and responses to self-report questionnaires. The sensor data were recorded locally on the devices during the experiments to prevent wireless packet loss. Participants provided self-reports after each experimental phase, utilizing established questionnaires like the Positive and Negative Affect Schedule (PANAS) and Self-Assessment Manikins (SAM). These ground truth values facilitate the correlation of physiological data with states of stress, amusement, and neutrality.

The LaborCheck dataset [35] contains information gathered from both computer monitoring (keyboard, mouse, and operating system usage) and wearable monitoring (physical activity, HR, and sleep patterns). Each participant’s data is organized into separate files, comprising 51 features along with labels that indicate stress levels based on self-reports. Computer monitoring data were collected using a custom application called LaborCheck, which synchronized with the cloud every 20 min. Meanwhile, wearable monitoring data were obtained from a Fitbit device. This dataset has several advantages, including its multimodal nature, data collection under real-world conditions, and suitability for various ML applications.

The StressDetection dataset [36] for real-time stress detection using a smartphone is organized into two folders based on labels: “calm” and “stress.” The data consist of various modalities derived from raw sensor data, including information from the ACC, linear ACC, gravity, GYRO, magnetometer, ambient light sensor, game rotation vector, and metrics on how many times the screen was touched or the delete key was pressed. The collected data were recorded directly into the internal memory of the smartphone, ensuring that the system could function offline. Additionally, stress levels were assessed through self-evaluation.

The MoodAware dataset [37] includes extracted features from raw data, organized in separate files based on specific modalities. It contains statistical features along with labels that represent the participant’s mood or its level, which are derived from a questionnaire. Due to an imbalance in the collected dataset, the authors decided to randomly undersample additional instances from the relaxed class, which is the majority class.

The GLOBEM dataset [41] includes multimodal data, e.g., location tracking, phone usage (screen status), Bluetooth scans, and call logs, all collected through the mobile application AWARE Framework. Additionally, information about physical activities and sleep behaviors was obtained from Fitbit. Post-processing steps were applied, which included feature normalization and feature discretization.

The ADARP dataset [38] consists of scanned documents featuring calendars that indicate the days when participants consumed alcohol. It further includes information captured from Empatica E4, e.g., skin conductance or EDA, SKT, three-dimensional body acceleration (ACC-X, ACC-Y, ACC-Z), BVP, and HR. Lastly, “Final Phone Survey Stress Events” provides of structured qualitative interviews that assess daily alcohol consumption using a timeline follow-back calendar and help validate self-reported and physiological stress markers.

The EmpaticaE4Stress dataset [39] consists of 29 folders, each corresponding to a different participant. Within each folder, there are six comma-separated values (CSV) files that contain all the data recorded by the Empatica E4 device, including BVP, EDA, TEMP, HR, IBI, and ACC. Labels can be assigned based on the figures presented in the official paper, which indicate task periods or rest periods.

**Fig. 2:**
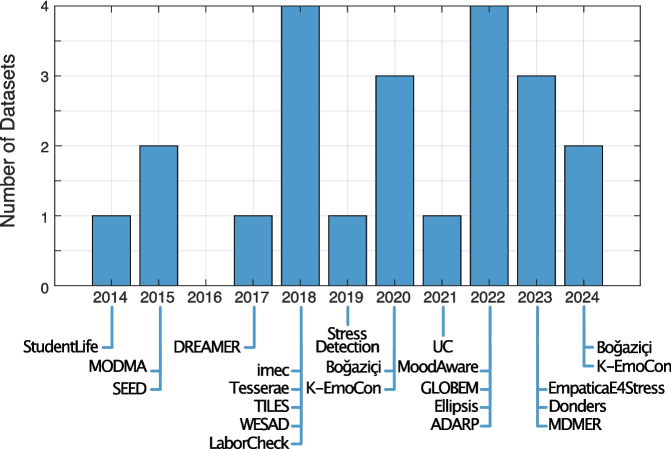
Datasets initial publication year distribution

**Fig. 3:**
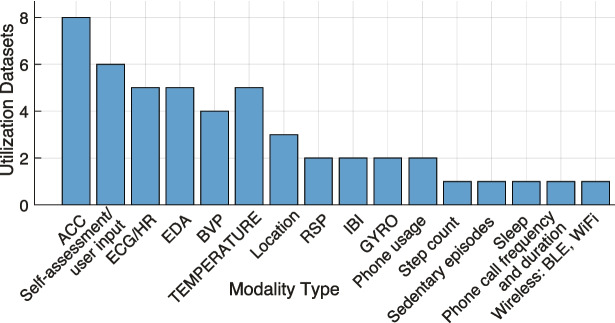
Distribution of modalities per use in datasets

The EmoWear dataset [40] was created using two wearable devices: the Zephyr BioHarness and the Empatica E4. After completing the data collection session, the raw data from all the wearable devices was transferred to a secure drive. The dataset is available in both CSV and Matrix file format (MAT) formats. Each participant has a dedicated folder containing 20 files, which include raw data from the devices, information about activities performed during the recording period, sift information, and labels derived from self-assessment surveys.

To summarize, most datasets comprise various collection methods, incorporating physiological measurements, behavioral assessments, self-reports, and environmental data. Figure 2 also encompass the distribution of datasets per year showing the growing interest of research community towards emotion recognition. This data is usually organized into folders or files either on a per-participant basis or according to specific data types. Many datasets utilize some wearables to record physiological data. Moreover, self-reported assessments are often integrated into the process, typically through standardized questionnaires, providing subjective data that can be correlated with objective metrics. There is a reliance on cloud-based and local storage solutions. Data is frequently stored in CSV file format, though other formats, e.g., MAT, are also employed depending on the dataset’s level of complexity. Post-processing techniques, such as feature normalization and discretization, were commonly applied to prepare these datasets for further analyses.

### Major Modalities

The analysis of the datasets shows the distribution of different modalities with a clear preference for certain types of data in emotion detection research (see Fig. 3). The most frequently cited modalities, e.g., ACC [33–40], self-assessment/user input [33, 34, 36, 38, 40], ECG/HR [34–36, 38, 40], and EDA [34, 37–40], reflect a strong focus on easily measurable, versatile, and widely applicable data sources. The use of ACC indicates its central role in activity and motion tracking, which are essential for various behavioral detection applications. Similarly, ECG/HR and EDA are integral for monitoring physiological responses, especially in studies investigating emotional states and stress, as well as overall health, making them fundamental in wearable health technology and biofeedback research. Self-assessment complements sensor data by providing subjective insights and is also widely used. This leads to the importance of combining objective and subjective measures in health and behavioral research.

Moderately collected modalities include SKT [34, 37–40], BVP [37–40], and location [33, 34, 41] receive fewer notices but remain significant in emotion recognition. BVP and SKT are crucial for specific health conditions, e.g., cardiovascular monitoring and temperature regulation, although they are less commonly used compared to more general-purpose sensors like ECG. Location, used for tracking movement, appears less frequently in datasets, likely due to its more specialized application in mobility and geographical studies rather than emotion recognition.

Lastly, modalities like step count and sedentary episodes and wireless communications [41] have lower usages counts, suggesting they are either emerging areas of research or serve more specific purposes, yet were collected as opportunistic ones. Besides, step count and sedentary behavior tracking were highlighted as important in studies on physical activity and lifestyle, but may not yet be as frequently utilized as direct physiological modalities, e.g., ECG or EDA. Wireless communication modalities, including Bluetooth and WiFi, are mostly present in the majority of modern handheld devices, but their research focus tends to be more remote from healthcare domain rather than related to emissions, addressing specific contexts, such as indoor positioning or environmental monitoring.

The highlighted distribution reflects both practical considerations and research trends. The high number of datasets counts of modalities like ACC, EDA, and ECG/HR highlight their utility in a broad range of applications, making them easier to collect and apply across various studies on emotion recognition. In contrast, modalities with fewer usages, e.g., location and BVP, may be used in research with specific objectives or face challenges related to data collection and integration.

### Data Collection Approaches

All identified open access datasets were collected following broadly similar methodologies, combining wearable devices, smartphone-based passive sensing, and self-reported assessments. However, each dataset included specific adaptations to address unique research objectives and participant demographics. Across studies, wearable devices, such as the Empatica E4, Fitbit, and RespiBAN, played a vital role in recording high-resolution physiological data. In contrast, smartphone applications were frequently used for behavioral monitoring, location tracking, app usage, and communication patterns, as seen in datasets like StudentLife [55] and GLOBEM [41]. This multimodal approach ensured comprehensive data collection in both real-world and controlled environments.

Certain datasets demonstrated unique features that extended the standard methodology. For example, WESAD [34] and EmoWear [40] detailed specific emotional or stress states using laboratory-based protocols involving mental arithmetic tasks or audiovisual stimuli. These controlled experiments were designed to create consistent and replicable emotional responses for ML models training. In contrast, datasets, such as GLOBEM [41] and the Moderate Depression Study [37] prioritized ecological validity by collecting data in free-living or clinical settings, leveraging longitudinal monitoring and real-time self-reported questionnaires. This approach captured individual variations from natural environments in emotional and physiological states.

Ethical considerations were universally adhered to, with all studies obtaining Institutional Review Board approval to ensure participant safety and data integrity. Recruitment strategies varied, from targeting specific clinical populations, such as individuals with medical conditions in the ADARP dataset [38], to broader academic populations, such as students and staff in StudentLife [55] and GLOBEM [41]. Certain datasets, like GLOBEM, explicitly oversampled underrepresented groups to enhance demographic diversity, a step that reflects growing awareness of inclusiveness in behavioral research.

Data processing and storage practices were also standardized but incorporated the dataset specifics. Preprocessing included filtering physiological signals, anonymizing sensitive data, and synchronizing multimodal streams. Storage systems were secured using encryption and were GDPR—or Health Insurance Portability and Accountability Act (HIPAA)—compliant, as demonstrated in datasets like WESAD [34] and the MoodAware [37]. Longitudinal studies, such as GLOBEM [41] introduced additional challenges, such as data retention policies and managing participant withdrawal requests.

**Table 2:** Population characteristics of analyzed datasets

Dataset and link to Table 1	Ref.	Participants	Age, years	Gender	Population type
StudentLife See details	[46]	48	n/a	10 females, 38 males	Students from various academic levels
WESAD See details	[47]	15	24–31; mean 27	Male and female (numbers unspecified)	Healthy young adults
LaborCheck See details	[63]	n/a	n/a	n/a	Desk-job workers in three organizations (INEEL, INE, and CENIDET)
StressDetection See details	[50]	63	19-65	38 females, 25 males	Individuals with depressed mood
MoodAware See details	[44]	14	Mean 21.6 (±2.8)	10 females, 4 males	University students with moderate depression
GLOBEM See details	[51]	497	n/a	n/a	University students and staff (Carnegie-classified R-1 university)
ADARP See details	[49]	n/a	n/a	n/a	AUD-diagnosed individuals undergoing treatment
EmpaticaE4Stress See details	[53]	29	20+	n/a	n/a but matching real work environment
EmoWear See details	[54]	48	20–30	12 males, 8 females	University students and staff

Combined data with Table 1 is available in Appendix 2

In summary, while the datasets followed a cohesive framework for data collection, processing, and storage, they were tailored to their specific contexts through variations in experimental setups, recruitment strategies, and data handling practices. These commonalities and differences highlight the evolving methodologies in stress, emotion, and mental health research, ensuring both scientific rigor and ethical compliance.

### Demographic and Other Coverages

The analyzed datasets have a strong focus on university students, young adults, and specific populations, such as diagnosed individuals (see Table 2). While these datasets offer valuable insights for targeted research, they inherently suffer from representational biases and a lack of demographic diversity.

First, the datasets are closely skewed toward younger populations, particularly university-affiliated individuals. This makes it challenging to generalize the findings to other demographics, such as the elderly, adolescents, or children. Furthermore, several datasets either omit ethnicity data or fail to ensure a diverse representation, potentially limiting the cross-cultural applicability of the research. The gender representation is uneven in many cases, with some datasets having a majority of male or female participants, while others provide no gender information at all. This lack of gender balance could influence the accuracy and fairness of models trained on these datasets.

Another significant limitation is the small sample sizes in many datasets, such as WESAD (15 participants) or Moderate Depression Study (14 participants). These small groups reduce statistical power and make it harder to derive reliable and generalizable insights. Finally, many datasets fail to incorporate detailed demographic details, such as socioeconomic status or specific health conditions, which are crucial for understanding broader behavioral patterns.

Finally, combined population characteristics of analyzed datasets (Table1) along with general information of those (Table 2) are provided in Appendix B.

Addressing these gaps through a more inclusive recruitment, balanced demographics, and larger participant pools would significantly enhance the robustness and applicability of future research outcomes.

## Data Collection/Processing Challenges and Identified Proposed Solutions

Emotion-related multimodal datasets for stress and emotion recognition often face challenges from complex human behavior, data variability, and sensor limitations. Based on the related papers, this section attempts to systematize common challenges and solutions from various studies. A summary of mentioned data-related challenges and solutions is detailed in Table 3.

**Table 3:** Summary of data-related identified challenges and proposed solutions

Challenge	Ref.	Proposed solution
Subjectivity and variability in self-reports	[55]	Used monotonic analysis, e.g., Spearman correlation, instead of linear correlation to improve data interpretation
	[68]	Suggested personalized models incorporating baseline surveys and daily session-based questionnaires to reduce subjectivity
Class imbalance in datasets	[70]	Minority Class Oversampling and Majority Class Undersampling are used
	[75]	Applied SMOTE for minority class oversampling
	[55]	The data were resampled using technique SMOTE
	[68]	Randomly undersampled majority class in imbalanced self-reported ground truth labels
Noise and motion artifacts in sensor data	[39]	Filtered PPG signals using Chebyshev II and Gaussian low-pass filters
	[70]	Applied Butterworth low-pass filters and normalization for consistent EDA signals
	[68]	A pre-processing unit with SVM classifier detects and removes the artifacts by analyzing skin temperature, accelerometer, and skin conductance signals
Synchronization and validation of sensor data	[40]	Achieved synchronization using jump-based events and adjusted ACC and GYRO coordinates; validated signal quality with SNR metrics and correlation analysis
Limited dataset	[55]	Suggested including sleep activity data
	[70]	Recommended extending datasets to diverse populations
	[41]	Missing values are omitted during analysis and use a median-based imputation
	[68]	Utilization of multimodal in terms of the collected data
Motion adaptation and environmental noise	[62]	The EDA signal was recorded on the rectus abdominis, the TEMP sensor was placed on the sternum
	[40]	Noise-canceling wireless headphones were utilized
	[75]	Techniques for data pre-processing were applied to obtain high-quality mining results
Generalization in modeling	[41]	A pre-text shuffling and reordering task offered to push the model to learn more generalizable representations
	[64]	Used gain ratio for feature selection to improve model performance
	[55]	Application of feature selection method
Data loss in transmission	[62]	The data could be stored locally and transmitted after the experiment

### Subjectivity and Variability in Self-reports

Self-reports remain inherently subjective. Respondents may interpret Likert scale values differently, introducing variability in the data and reducing reliability. For example, the StudentLife dataset [55] highlights the difficulty of ensuring consistency in perceived response values among participants, which affects the assessment of mental health severity scores. To address this, the researchers employed monotonic analysis using Spearman rank-order correlation rather than linear correlations to improve data interpretation [55]. Personalized stress and emotion models have also been suggested, incorporating baseline surveys and daily session-based questionnaires to mitigate bias caused by subjectivity [68].

### Class Imbalance in Datasets

Class imbalance is a recurring issue in real-world datasets where certain emotional states or stress conditions are underrepresented. For example, the ADARP dataset reported a significant imbalance in stress events due to the difficulty of manufacturing stress stimuli in real-life settings [70]. To resolve this, techniques like SMOTE and majority class undersampling were employed to create balanced datasets [55, 70, 75]. Similarly, undersampling of the majority class was utilized to address imbalances in self-reported ground truth labels [68].

### Noise and Motion Artifacts in Sensor Data

Motion artifacts and environmental noise significantly degrade the quality of physiological signals, such as EDA and PPG. In the dataset proposed in [39], researchers filtered raw PPG signals using Chebyshev II and Gaussian low-pass filters to minimize noise and artifacts. Similarly, the ADARP dataset employed Butterworth low-pass filter and normalization to ensure consistency in EDA signals [70]. Preprocessing techniques to detect and remove motion artifacts using ACC were also applied in the dataset proposed in paper [68].

### Synchronization and Validation of Sensor Data

Synchronizing data from multiple wearable devices and validating its quality is a challenging task. In the EmoWear dataset, synchronization was achieved by asking participants to perform three consecutive jumps, aligning sensor readings based on these events [40]. Signal quality was evaluated using SNR metrics and correlation analysis, ensuring the reliability of the collected data. Additionally, in order to match the coordinate system of the trunk-worn devices, the authors applied adjustments to the accelerometers and gyroscopes.

### Limited Dataset

Many datasets have limited scope regarding sample size, participant diversity, or feature variables. For example, the StudentLife dataset lacked sleep activity data, while ADARP primarily included undergraduate students, reducing generalizability [55, 70]. Suggested improvements include expanding datasets by diversification of populations and additional features, such as sleep quality and social networking data. Advanced feature extraction techniques and median-based imputation methods were also proposed for handling missing or limited data [41]. Another problem raising concerns is a possible failure of a single sensor-based system. To address this challenge, Can et al. [68] suggested collecting multimodal data, implementing robust pre-processing, and integrating feature extraction modules to ensure the reliability of the system.

### Motion Adaptation and Environmental Noise

Another challenge is adapting sensor setups to allow free movements of participants without compromising data quality. The WESAD dataset utilized innovative sensor placements, e.g., recording EDA signals from the abdomen and placing temperature sensors on the sternum, to enhance data reliability during movement [62]. To mitigate environmental noise, noise-canceling headphones were employed in the EmoWear dataset during experiments involving emotionally eliciting video clips [40]. Also, a similar issue was mentioned in [75], where the authors proposed techniques for data pre-processing to handle this problem. Nonetheless, motion artifacts remain a significant technical challenge, as noise from movement can significantly impact the performance of wearable biosensors [39, 76].

### Generalization in Modeling

Domain generalization techniques often struggle with over-fitting and fail to perform well across datasets. In the GLOBEM dataset, researchers suggested designing pre-test tasks, e.g., predicting the next behavior feature value, to improve the generalizability of models across domains [41]. Another technique that can improve the capability for generalization is feature selection. For example, the authors of [64] applied a gain ratio feature selection algorithm to rank and select the most effective features. Also, the work [55] applied feature selections to improve the performance of the models.

### Packet Loss

Packet loss during data transmission is also a potential challenge in wearable sensor-based studies, especially in scenarios requiring wireless communication. This issue can result in incomplete datasets, compromising the reliability of the analysis. To mitigate this, some works opted for local data storage on wearable devices. For example, the WESAD dataset stored all recorded data locally on the devices and transferred it to a computer for further processing after the experiments were completed, which ensured no data was lost due to transmission errors [62]. Combining local storage with occasional batch uploads when stable connections are available for future research could further enhance data reliability and collection efficiency.

Notably, the challenges listed above are identified and summarized from the corresponding works, while more may remain hidden as obvious from the data science perspective.

## Outlined Limitations of the Datasets

Apart from technical challenges, the included studies also highlighted other limitations associated with data collection via wearable technologies for mental health monitoring.

One primary limitation lies in the variability in daily life conditions. Since individuals live in unique environments, engage in various activities, and possess different routines and personal habits, it might be difficult to identify emotions on a general scale precisely. As a result, these personal variants may introduce inconsistencies and noise into the data [39]. To mitigate this, incorporating contextual data, such as user activity, location, and environmental factors, can enhance the accuracy of emotion recognition models by accounting for the context of data collection. However, the majority of the datasets are based on data collection in laboratory environments or applied video/audio records to force participants to respond with the anticipated emotion. This point is critical since it may not correspond to real-world conditions. In fact, many works focus on HR, EDA, ACC, RESP, and SKT, but not video or audio [40]. It becomes necessary to explore the potential utilization of emotion detection in an unobtrusive manner in users’ natural environments. However, to date, most of the research on emotion detection has been done in laboratory settings. By gathering individual physiological data and tracking patterns of physical activities, it becomes possible to improve the accuracy of emotion detection, especially by integrating self-assessment as a crucial additional measurement metric. This approach enables models to better handle variability and make more accurate predictions [37, 59]. Advanced signal processing techniques, e.g., noise reduction and signal smoothing, can also improve data quality and emotion detection accuracy in real-world settings [77]. Moreover, selecting the optimal combination of physiological modalities is critical, and polynomial-time modality selection algorithms have been developed to address this challenge [38].

Another limitation lies in the high variability of emotion responses, e.g., stress responses vary significantly among individuals [78] due to factors, such as genetics, lifestyle, and psychological condition, complicating the development of a universal stress detection model. Personalized ML models that adapt to individual differences can improve prediction accuracy by tailoring the model to individual data, thereby providing more reliable stress detection [72, 79]. For example, the use of a self-organizing map (SOM) for personalized stress detection has shown the potential to adapt to individual differences [34]. Additionally, a layered system architecture for personalized stress monitoring using low-cost, easy-to-wear PPG devices has been developed [80].

Notably, the majority of the datasets suffer from limited populations (see Table 2). Although training on limited number of participants can lead to an over-fitting problem [62, 67], which means the trained models will not be able correctly predict for others, it is indisputable that introducing datasets that are useful for design and development of ML/DL models and proposed approaches can achieve impressive results [7, 23, 72, 79]. One way to overcome the above-mentioned challenges is extending pre-existing datasets and merging data from similar devices, e.g., EEG and phone usage logs [40, 62, 66].

Implementing robust data encryption and anonymization techniques can be used to protect sensitive data privacy to fulfill existing regulations [30]. Ensuring secure data storage and transmission, as well as removing personal identifiers, are crucial measures to address privacy risks [33]. As another countermeasure, Table 1 emphasizes that many sources are governed by strict terms and conditions. For example, data collectors of the MODMA, SEED, and K-EmoCon datasets reserve the rights to modify their respective agreements at any time. Users will be informed of changes and given the option to opt out, which would render the agreement void and revoke access to the dataset. For MODMA, explicit participant consent is required for using their data in publications, with consent information included in the participant questionnaire file [56]. Most datasets originating from the USA limit the use strictly to the research described in the agreement, and the covered entity must be included in the authorship of related publications [44, 57], similarly to what the AIA emphasizes [28]. Users are required to fully compensate and protect dataset providers from any losses or damages arising from misuse or breaches of the agreement, covering all legal costs and damages. These conditions ensure ethical usage and proper acknowledgment but impose significant compliance requirements on users. For example, some authors request all of their own papers related to the datasets be cited if the dataset is used in new research.

It is necessary to emphasize that, although long-term studies are vital for understanding chronic conditions and emotional patterns over time, they present additional challenges. These include participant fatigue, device wearability issues, and the need for consistent data maintenance. From the data processing aspect, these challenges can be addressed by developing user-friendly wearable devices that are comfortable, unobtrusive, and capable of functioning for extended periods without frequent maintenance [19]. Ensuring continuous and accurate data collection in real-world settings is also critical. The use of smartphone sensors for unobtrusive monitoring and passive sensing can assist in achieving this goal [81, 82]. In high-stress, fast-paced work environments, wearable sensors capable of real-time monitoring have been employed to classify stress episodes by comparing baseline data with periods preceding self-reported stress episodes [83]. Moreover, the majority of prior research performed binary classifications or a limited set of class labels of unseen instances using ML-enabled models. However, human states of stress are far more complex, particularly when accounting for factors derived from real-life situations [84].

Symptom variability, where stress and other emotional symptoms can differ significantly among individuals and over time, complicates the development of accurate ML models for detection. Multimodal data collection, which combines various sources, such as physiological signals, behavioral data, and self-reports, can provide a more comprehensive understanding of emotional states [85, 86]. It can be presented differently across individuals, focusing on the most relevant physiological markers through feature selection methods can help manage symptom variability and improve detection accuracy [87]. Robust feature extraction techniques have been developed to identify key indicators of depressive symptoms from longitudinal data [88]. In addition, emotional stimuli variability is another challenge. Different stimuli can elicit different emotional responses, making it difficult to develop models that generalize well across all emotional triggers. Including a diverse range of stimuli during data collection can enhance model generalizability and improve the accuracy of emotion recognition across different contexts [89].

Next, the subjective nature of stress and the complex issues linked to balancing datasets are examples of additional constrains. These problems can be addressed by modeling time variation in stress labels and using iterative search algorithms to determine optimal input lengths around stress events. Datasets can be balanced by applying majority undersampling and minority oversampling techniques [38]. Efficient management and cleaning of large volumes of multimodal data are also essential. Tools for efficient data collection and integrity checks have been also developed to reduce data cleaning time [90].

Finally, the evolving regulatory landscape, particularly the EU’s AIA [27] and GDPR [30], could significantly impact the analysis of existing open access emotion-related datasets. Stricter transparency obligations [28] would require dataset providers to clearly disclose data collection methodologies, sources, and consent mechanisms, ensuring compliance with informed consent standards. Limitations in decision-making based solely on biometric data [29] may restrict how these datasets can be used in predictive modeling or automated emotion classification without human oversight. Additionally, regulations emphasizing automated consent management could impose stricter requirements on dataset usage, potentially requiring retroactive consent verification or restricting access to datasets lacking explicit user consent. These measures could lead to reduced availability of open access emotion datasets or require substantial modifications to align with legal and ethical standards, thereby potentially affecting ongoing research and development in emotion recognition technologies.

All things considered, these limitations underscore the complexities of using wearable devices for stress and mental health monitoring and highlight the ongoing need for technological and methodological advancements to address these issues effectively.

## Paper Summary

Rapid advancement of wearable technologies and mobile sensors has significantly boosted emotion recognition and mental health monitoring. This study reviewed the available open access emotion-related datasets, focusing on their unique characteristics, collection methods, and the challenges associated with working with emotion-related data.

This paper acts as a cornerstone for developing accurate, ethical, and practical emotion recognition technologies by offering an in-depth analysis of critically rare-to-find and impactful datasets. These systematized insights could be invaluable for system developers, engineers, clinicians, researchers, and policymakers seeking to leverage emotion detection in diverse research and clinical applications.

## Abbreviations

 

*ACC*: Accelerometer
*AI*: Artificial intelligence
*AIA*: Artificial Intelligence Act
*AUD*: Alcohol use disorder
*BVP*: Blood volume pulse
*BLE*: Bluetooth low energy
*CNN*: Convolutional neural network
*CSV*: Comma-separated values
*DL*: Deep learning
*ECG*: ElectroCardioGraphy
*EDA*: Electrodermal activity
*EEG*: ElectroEncephaloGraphy
*EMA*: Ecological Momentary Assessment
*EMG*: Electromyography
*ER*: Emotion recognition
*ERS*: Emotion recognition system
*EU*: European Union
*fMRI*: Functional magnetic resonance imaging
*MAT*: Matrix file format
*GA*: Genetic algorithms
*GDPR*: General Data Protection Regulation
*GNSS*: Global Navigation Satellite System
*GSR*: Galvanic Skin Response
*GYRO*: Gyroscope
*HIPAA*: Health Insurance Portability and Accountability Act
*HR*: Heart rate
*HRV*: Heart rate variability
*IBI*: Inter-beat interval
*IMU*: Inertial measurement unit
*kNN*: *k*-nearest neighbors
*LSTM*: Long short-term memory
*ML*: Machine learning
*PAM*: Photographic affect meter
*PRISMA*: The Preferred Reporting Items for Systematic reviews and Meta-Analyses
*PANAS*: Positive and Negative Affect Schedule
*PPG*: Photoplethysmography
*SOM*: Self-Organizing Map
*RF*: Random forest
*RESP*: Respiration
*RNN*: Recurrent neural network
*RSP*: Respiration
*SCG*: Seismocardiography
*SAM*: Self-assessment manikins
*SNR*: Signal-to-noise ratio
*SKT*: Skin temperature
*SMOTE*: Synthetic Minority Oversampling Technique
*SVM*: Support vector machine
*TEMP*: Temperature
*WiFi*: Wireless fidelity

## Data Availability

The data set(s) supporting the results of this article is(are) available in repositories as specified in Table 1.

## Appendix A: Identification of studies from public databases and registers

To identify relevant datasets for this review, we conducted a structured search in three major open-access repositories: Zenodo, IEEE DataPort, and Kaggle. The mentioned sources are the most frequently used for publishing of datasets. The search focused on locating multimodal datasets related to emotion, stress, or mental health detection and recognition, particularly those incorporating physiological signals from wearable devices. Searches were conducted between June and August 2024 using the initial keyword combinations: “{{depression OR stress OR emotion OR anxiety OR mental} AND {data OR dataset}}.”

Where available, repository filters were applied to restrict results to the following:

- Open access or freely downloadable datasets
- Dataset-type entries only
- Publication/update date between 2015 and 2024

Each platform’s native search tools were used to query available metadata fields, such as titles, descriptions, and tags. Table 4 summarizes the execution of our search strategy across the three platforms. Notably, IEEE DataPort was not able to process the query (while Zenodo and Kaggle allowed for a systematic search), and thus, the keywords were inserted sequentially.

**Table 4:** Utilized dataset filters

Platform	Available filters	Dataset type and date range
Zenodo	Title, Keywords, Access status, Resource types	Resource types: Dataset; Access status: Open, Restricted
IEEE DataPort	Title, Abstract, Keywords, Dataset type, Categories	Dataset type: Open Access; Keywords: <None>; Machine Learning, Deep Learning; Categories: Artificial Intelligence, Biophysiological Signals, Health, IoT, Machine Learning, Signal Processing; Dataset only, 2015–2023
Kaggle	Titles, Tag	Tag: Datasets; Datasets only, updated after 2015

The initial screening produced more than 3000 dataset entries spanning far beyond the scope of multimodal emotion recognition research. Datasets were excluded if:

- Not related to the scope of research (search engine imperfections)
- Lacked full open access (e.g., required email request or links were non-functional)
- Included only a single modality (e.g., fMRI-only)
- Were outdated or no longer actively maintained (e.g., before 2015)

We excluded datasets that solely used surveys or lacked physiological/contextual signals, which factually resulted in only 34 datasets. Further, we conducted a structured in-depth review and added further classification based on three key axes:

- Inclusion was limited to datasets incorporating physiological or contextual data from wearables (e.g., smartwatches, wristbands) or mobile devices (e.g., smartphones).
- We included datasets that target psychological states related to emotion, such as stress, depression, or affect, regardless of whether they use binary, ternary, or dimensional labels (e.g., valence-arousal).
- Datasets must be open access or available upon request. Datasets requiring payment, institutional partnerships, or with unresponsive contacts were noted but not excluded.

Following the exclusion process, 18 datasets were retained. An additional 4 datasets were included based on extended searches or related works from the same dataset contributors, and the search query used was refined to “{{depression OR stress OR emotion OR anxiety OR mental} AND {detection OR classification} AND data*}” with filters following those from Table 4. The final set of 22 datasets reflects recent, multimodal, and relevant data sources to emotion and stress detection using wearable technologies. Notably, only 9 of them are fully accessible.

As of the systematic output, Table 1 presents all reviewed datasets (as of September 2024) with references, modality descriptions, emotional target classes, and accessibility status. This format enables comparative analysis across datasets for model transferability, sensor selection, and coverage of emotional categories. Moreover, we denoted access levels with the following symbols:

- $\checkmark$: freely and publicly accessible
- $\looparrowright$: partially available or accessible via request form
- : restricted or unclear terms of use
- : available only upon request by email
- : claimed accessible but unresponsive or unavailable in practice

Some datasets suffer from limited metadata or non-standardized emotional labels. In such cases, we relied on published papers and official dataset pages. Attempts were made to contact dataset authors when access was ambiguous;  denotes non-responsiveness.

## Appendix B: Extended data on fully open access datasets (Tables 1 and 2)

**Table 5:** Extended data on emotion-related fully open access datasets as of 08.09.2024

Dataset	Ref.	Participants	Age, years	Gender	Population type	Wearables/sensors	Types of emotions	Modalities
StudentLife	[46]	48	n/a	10 females, 38 males	Students from various academic levels	Smartphone	Depression, stress, loneliness	ACC, location (GNSS), phone call frequency and duration, self-assessment
WESAD	[47]	15	24–31; mean 27	n/a	Healthy young adults	Polar M600 smartwatches, smartphone (Android)	Binary stress classes	Lab: ECG, EDA, RSP, SKT, ACC; Field: IBI, HR, ACC, phone usage, GNSS, self-assessment
LaborCheck	[63]	n/a	n/a	n/a	Desk-job workers (INEEL, INE, and CENIDET companies)	Fitbit	Ternary: low, medium, high	HR, ACC
StressDetection	[50]	63	19–65	38 females, 25 males	Individuals with depressed mood	Smartphone	Binary stress classes	HR, phone usage, gravity, ACC, GYRO, self-assessment
MoodAware	[44]	14	Mean 21.6 (±2.8)	10 females, 4 males	University students with moderate depression	Empatica E4	Binary depression classes	BVP, EDA, ACC, SKT
GLOBEM	[51]	497	n/a	n/a	University students and staff (Carnegie-classified R-1 university)	Smartphone, Fitbit wrist worn	Binary depression classification	Location, phone usage (screen status), Bluetooth, call logs, WiFi, physical activities (step counts, sedentary episodes), sleep duration and quality
ADARP	[49]	n/a	n/a	n/a	AUD-diagnosed individuals undergoing treatment	Empatica E4	Binary stress classes	EDA, ACC, SKT, BVP, HR, user input on self-reported emotions and cravings
EmpaticaE4 Stress	[53]	29	20+	n/a	n/a but matching real work environment	Empatica E4	Stress levels	BVP, EDA, SKT, HR, IBI, ACC
EmoWear	[54]	48	20–30	8 females, 12 males	University students and staff	ST SensorTile.box, Zephyr BioHarness 3, Empatica E4	Valence, arousal, and dominance	ACC, GYRO, ECG, BVP, RSP, EDA, SKT, self-assessment

**Acronyms**: *EEG* electroencephalography, *ECG*electrocardiography, *BVP* blood volume pulse, *SKT* skin temperature (Skin Temperature (SKT) and Temperature (TEMP) merged for simplicity), *ACC* accelerometer, *GYRO* gyroscope, *PPG* photoplethysmography, *RSP* respiration, *IBI* inter-beat interval, *HR* heart rate, *GNSS* Global Navigation Satellite System, *HRV* heart rate variability, *EDA* electrodermal activity and*GSR* galvanic skin response refer to the same but are kept separately
